# Impact of Systematic Factors on the Outbreak Outcomes of the Novel COVID-19 Disease in China: Factor Analysis Study

**DOI:** 10.2196/23853

**Published:** 2020-11-11

**Authors:** Zicheng Cao, Feng Tang, Cai Chen, Chi Zhang, Yichen Guo, Ruizhen Lin, Zhihong Huang, Yi Teng, Ting Xie, Yutian Xu, Yanxin Song, Feng Wu, Peipei Dong, Ganfeng Luo, Yawen Jiang, Huachun Zou, Yao-Qing Chen, Litao Sun, Yuelong Shu, Xiangjun Du

**Affiliations:** 1 School of Public Health (Shenzhen) Sun Yat-sen University Guangzhou China; 2 School of Intelligent Systems Engineering Sun Yat-sen University Guangzhou China; 3 Lingnan College Sun Yat-sen University Guangzhou China

**Keywords:** COVID-19, new cases, growth rate, multidimensional factors, statistical machine learning

## Abstract

**Background:**

The novel COVID-19 disease has spread worldwide, resulting in a new pandemic. The Chinese government implemented strong intervention measures in the early stage of the epidemic, including strict travel bans and social distancing policies. Prioritizing the analysis of different contributing factors to outbreak outcomes is important for the precise prevention and control of infectious diseases. We proposed a novel framework for resolving this issue and applied it to data from China.

**Objective:**

This study aimed to systematically identify national-level and city-level contributing factors to the control of COVID-19 in China.

**Methods:**

Daily COVID-19 case data and related multidimensional data, including travel-related, medical, socioeconomic, environmental, and influenza-like illness factors, from 343 cities in China were collected. A correlation analysis and interpretable machine learning algorithm were used to evaluate the quantitative contribution of factors to new cases and COVID-19 growth rates during the epidemic period (ie, January 17 to February 29, 2020).

**Results:**

Many factors correlated with the spread of COVID-19 in China. Travel-related population movement was the main contributing factor for new cases and COVID-19 growth rates in China, and its contributions were as high as 77% and 41%, respectively. There was a clear lag effect for travel-related factors (previous vs current week: new cases, 45% vs 32%; COVID-19 growth rates, 21% vs 20%). Travel from non-Wuhan regions was the single factor with the most significant impact on COVID-19 growth rates (contribution: new cases, 12%; COVID-19 growth rate, 26%), and its contribution could not be ignored. City flow, a measure of outbreak control strength, contributed 16% and 7% to new cases and COVID-19 growth rates, respectively. Socioeconomic factors also played important roles in COVID-19 growth rates in China (contribution, 28%). Other factors, including medical, environmental, and influenza-like illness factors, also contributed to new cases and COVID-19 growth rates in China. Based on our analysis of individual cities, compared to Beijing, population flow from Wuhan and internal flow within Wenzhou were driving factors for increasing the number of new cases in Wenzhou. For Chongqing, the main contributing factor for new cases was population flow from Hubei, beyond Wuhan. The high COVID-19 growth rates in Wenzhou were driven by population-related factors.

**Conclusions:**

Many factors contributed to the COVID-19 outbreak outcomes in China. The differential effects of various factors, including specific city-level factors, emphasize the importance of precise, targeted strategies for controlling the COVID-19 outbreak and future infectious disease outbreaks.

## Introduction

A new pneumonia disease emerged and was later named COVID-19 [1]. COVID-19 is caused by the novel SARS-CoV-2 and has become a major global health threat [2,3]. Due to massive population movement during the early stage of the COVID-19 epidemic, the disease rapidly spread in China. To keep the spread of COVID-19 in control, the Chinese government implemented rapid and strict intervention measures, such as quarantining Wuhan and its surrounding cities in the Hubei Province on January 23 and 24, 2020, banning public transportation, cancelling activities involving a gathering of people, extending the Spring Festival holiday, postponing the opening of schools, and setting up monitoring, testing, and isolation policies [4,5]. It has been proven that these strong measures have effectively slowed down, and even prevented, the spread of COVID-19 in China. The total number of new cases across the country has decreased rapidly and has been kept at a low level in the past several months [6,7].

The spread of COVID-19 depends on many factors. The human population is naïve to SARS-CoV-2, but there is currently no evidence showing dramatic changes in the virus [8]. Until now, many studies based on both statistical and mechanistic models have explored and confirmed the effect of population movement on the spread of COVID-19 [9-11]. Socioeconomic status, climate conditions, and intervention measures vary among cities across China. As a result, regional systems, resources, and the country’s capacity for responding to public health risks and events are directly linked to the outcomes of an outbreak [12]. Moreover, the allocation of medical resources and other related factors, such as the number of hospital beds, have a positive effect on the control of the epidemic [13,14]. In addition, many socioeconomic factors, such as population number, population density, and social activities, mediate the spread of the disease [15]. Studies have confirmed that environmental factors influence the seasonal transmission of pathogens, but the effect of environmental factors on COVID-19 is still controversial [16-19]. Furthermore, while previous upper tract respiratory infections might relate to infections by human coronaviruses and provide some cross-protection against SARS-CoV-2, the relationship between previous upper tract respiratory infections and COVID-19 is unclear and must be further studied [20]. Therefore, although the integrated effects of several influencing factors on the outbreak outcomes of COVID-19 have been analyzed [21], a comprehensive analysis has yet to be conducted.

We collected a comprehensive dataset and used a correlation analysis and machine learning algorithm to identify and assess national-level and city-level contributing factors to the outbreak outcomes of COVID-19 in China.

## Methods

### Data

#### Data Collection

As of March 1, 2020, the number of new COVID-19 cases in most prefecture-level cities in China has declined to 0. Therefore, data from 343 prefecture-level cities in China from January 17 to February 29, 2020 were collected and used in this study. This period was also the main COVID-19 outbreak period in China. Data were evaluated on a weekly scale comprised of the following 6 weeks: week 1, January 17-23; week 2, January 24-30; week 3, January 31 to February 6; week 4, February 7-13; week 5, February 14-20; and week 6, February 21-29. For each week, cities with 0 new cases were excluded from further analysis. Weekly new cases and COVID-19 growth rates were considered response/dependent variables, and a variety of regressors/independent variables were included in this study. These independent variables were divided into the following 6 categories: travel-related (current week), travel-related (previous week), medical, socioeconomic, environmental, and influenza-like illness (ILI) variables (Table S1 in Multimedia Appendix 1).

#### New Cases and COVID-19 Growth Rates

Confirmed COVID-19 case data were downloaded from daily official reports from the health commission, and weekly accumulative new case data were extracted. A proxy measurement for the reproductive number (R_proxy_) was used as the indicator for COVID-19 growth rate, which was defined as the number of new cases in the following week normalized by the number of new cases in the current week [22], as follows:


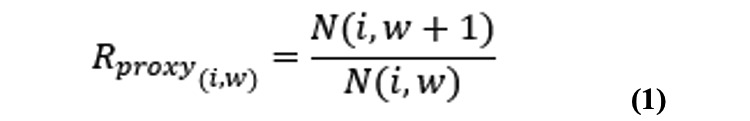


In this equation, w denotes week, ranging from 1-6, N is the number of weekly cumulative new cases, and i represents the ith city.

#### Travel-Related Factors

The daily domestic population movement data were derived from Baidu Qianxi [23]. The data are based on the positioning and transportation information systems of Baidu Location Based Services and Baidu Tianyan. The system collects location information that is voluntarily uploaded by users using Baidu services in real time and draws a population migration map based on global positioning system, Wi-Fi location, IP address, and signal tower information. We obtained city-level crowd movement information from this map for travel-related data. The following 5 city-level measurements were used in this study: population flow from Wuhan (Wuhan flow), population flow from Hubei, excluding Wuhan (Hubei/non-Wuhan flow), population flow from regions in mainland China, excluding Hubei (non-Hubei flow), population flow within a city (city internal flow), and the activity intensity within a city (city internal flow index), which was obtained by normalizing the population flow within a city by its population. Weekly measures were obtained based on the sum of daily data. Due to the latent period of COVID-19, travel-related factors from the previous week were considered separate from travel-related factors from the current week in this study. Hubei/non-Wuhan flow and non-Hubei flow measurements were combined to obtain non-Wuhan flow measurements, and city internal flow and the city internal flow index were combined to obtain city flow measurements.

#### Medical Factors

The number of doctors, hospitals, beds, and outpatients and emergency patients were derived from the 2018 edition of the China Health & Family Planning Statistical Yearbook [24]. Only province-level data were available for outpatient data, so city-level values were obtained in proportion to each city’s population. Data for the number of COVID-19 treatment hospitals were extracted from announcements issued by provincial health committees. Values were kept the same across all 6 weeks for each city.

#### Socioeconomic Factors

Socioeconomic factors, including population number, population density, gross domestic product, per capita income, and percentage of the population aged ≥65 years were derived from the 2018 China City Statistical Yearbook [25]. Except for the percent of the population aged ≥65 years, all socioeconomic data were at the city level. Province-level data for the percent of the population aged ≥65 years were used for each city. Values were kept the same across all 6 weeks for each city.

#### Environmental Factors

The daily climate data for each city, which included the highest temperature, lowest temperature, average temperature, relative humidity, and absolute humidity were downloaded from the China Meteorological Administration website [26]. Weekly data were calculated by averaging the daily data.

#### ILI factors

The average percentage of ILI occurrences for each city from 2016 to 2018 were calculated based on weekly report data from the Chinese National Influenza Center. The data were based on the number of samples tested in 554 sentinel hospitals in 31 provinces in China.

### Correlation Analysis

Spearman correlation was used to evaluate the relationship between a single factor and either the number of new cases or COVID-19 growth rates. A significance level of .05 was used in this study.

### Machine Learning Framework

#### Feature Selection and Feature Importance

First, a nonlinear regression tree model, which was made with XGBoost framework (extreme gradient boosting) [27], was used to fit weekly new cases and COVID-19 growth rates. Next, sequential backward floating selection was iterated to train the XGBoost [28,29] model to obtain the final model by minimizing the mean squared error. Sequential backward floating selection is a sequential feature selection method based on a greedy search algorithm. It removes features one by one from the full set of features and evaluates the error function. When the error reaches the optimum level, the combination of left-over features is regarded as the optimal feature combination. Finally, the importance of each selected factor was determined based on the number of times the factor was split by the tree model, which was determined by XGBoost. For each week, a contribution percentage was calculated for each factor based on its relative importance [30].

#### Shapely Additive Explanation Analysis

Shapley additive explanation (SHAP) [31] is an interpretable method for analyzing the output of machine learning models. SHAP analysis was used to calculate the contribution of selected factors for each city and week. A predicted value (ie, SHAP value) was generated for each data sample, and the value was uniformly assigned to each feature of the sample. The following equation shows how the predicted value of the machine learning model was calculated:

y_i_ = y_base_ + f(x_i1_) + f (x_i2_) + … + f(x_iN_)        **(2)**

In this equation, the ith sample was defined as x_i_, the jth feature of the ith sample was defined as x_ij_, the predicted value of the machine learning model for the sample was y_i_, and the reference value of the model (ie, the mean value of the target sample variable) was defined as y_base_. Furthermore, f(x_ij_) was the SHAP value of x_ij_. f(x_ij_)>0 indicates that the feature increases the predicted value and has a positive contribution; otherwise, the feature reduces the predicted value and has a negative contribution.

SHAP package [32] was used to calculate a marginal contribution value (ie, SHAP value) based on the trained XGBoost model [33-35]. The SHAP value was used to measure the contribution of different important factors for each city.

## Results

### Single-Factor Analysis

**Figure 1:**
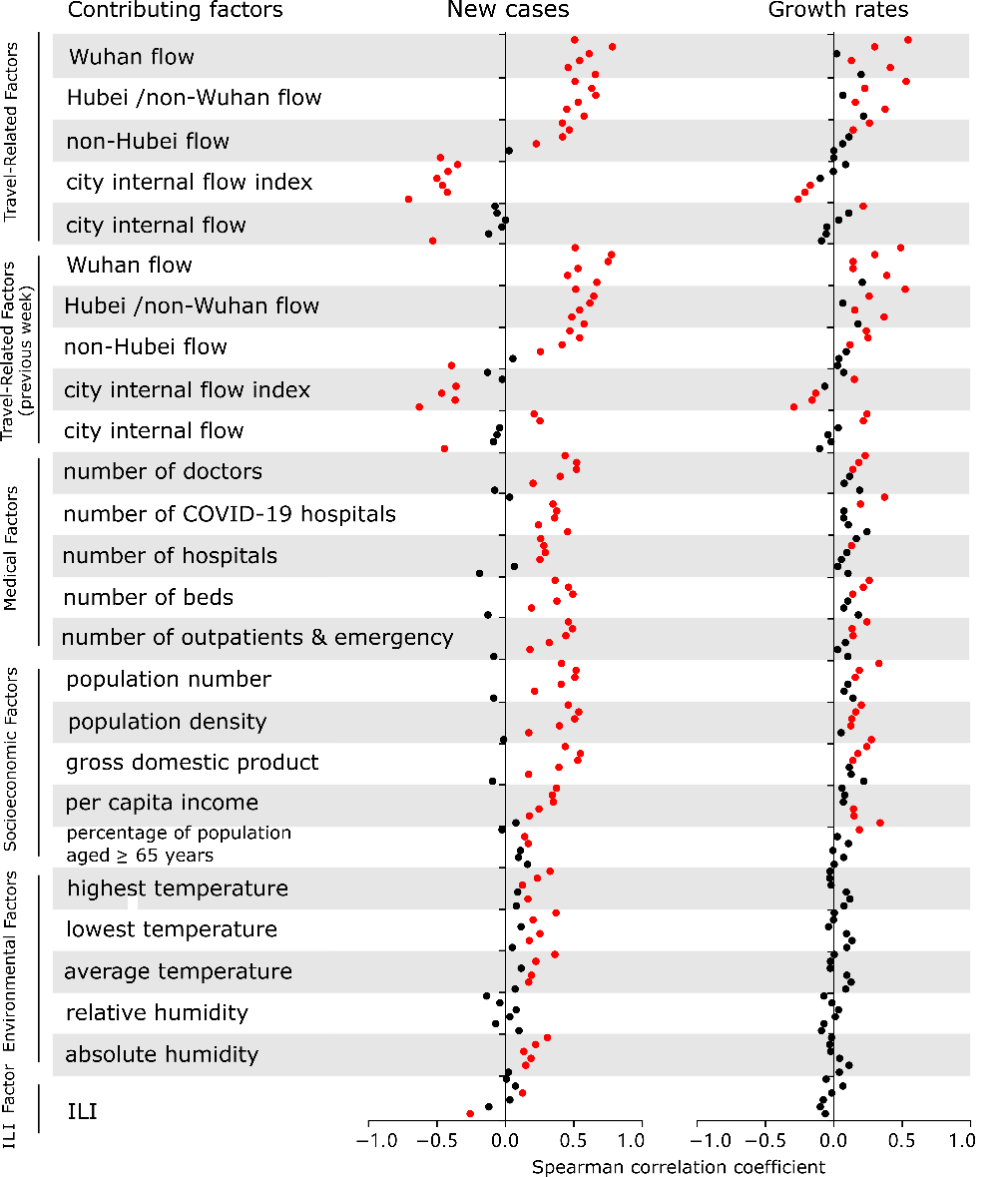
Correlation analysis of contributing factors for new COVID-19 cases (left) and COVID-19 growth rates (right). Dots from the top to the bottom for each factor indicate the corresponding Spearman correlation coefficient for 6 consecutive weeks (January 17 to February 29, 2020). Significant correlations are colored in red. ILI: influenza-like illness.

Based on the single-factor Spearman correlation analysis, many factors significantly correlated with both the number of new cases and COVID-19 growth rates in China (Figure 1). All factors significantly correlated with the number of new cases during at least 1 week, except relative humidity. Similarly, only travel-related (current and previous weeks), medical, and socioeconomic factors significantly correlated with COVID-19 growth rates during at least one week, while environmental and ILI factors did not correlate with COVID-19 growth rates (Figure 1). Wuhan flow, Hubei/non-Wuhan flow, and their corresponding measures in previous weeks positively correlated with the number of new cases during all 6 weeks, while the city internal flow index negatively correlated with the number of new cases during all 6 weeks. Many factors significantly correlated with the number of new cases for at least 5 weeks, but only Wuhan flow from the previous week and population density significantly correlated with COVID-19 growth rates for at least 5 weeks (Figure 1).

### Multifactor Analysis Based on the Machine Learning Algorithm

**Figure 2:**
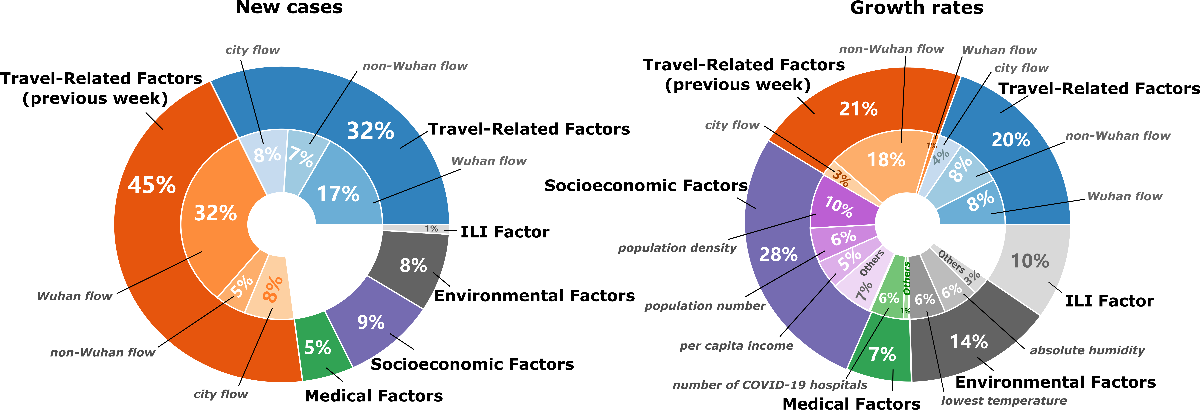
Contribution of various factors to new COVID-19 cases and COVID-19 growth rates in China. ILI: influenza-like illness.

Our additional analysis showed that many factors correlated with each other (Figure S1 in Multimedia Appendix 2). As a result, a nonlinear method was needed to assess the contribution of correlated factors on the spread of COVID-19 in China. Machine learning methods are good for solving nonlinear problems, and we used XGBoost [28,30] to create a nonlinear regression tree model for this study. Important factors were selected based on the cross-validation procedure in the XGBoost framework. Figure S2 in Multimedia Appendix 2 shows the number of factors selected for the final model. No single factor was consistently selected for all 6 weeks, but the overall contribution percentage could be obtained (Figure 2, Multimedia Appendix 2). Figure 2 shows that travel-related factors for the current week (contribution: new cases, 32%; COVID-19 growth rates, 20%) and related measures from the previous week (contribution: new cases, 45%; COVID-19 growth rates, 21%) were the main factors that drove changes in the number of new cases and COVID-19 growth rates, with total contribution percentages of 77% and 41%, respectively. For new cases, the contributions of other factors were no more than 10%. For COVID-19 growth rates, socioeconomic factors were also important, and they had a combined contribution percentage of 28%. Other factors also contributed to COVID-19 growth rates in China, with environmental, ILI, and medical factors contributing 14%, 10%, and 7%, respectively. For new cases, the leading individual factors with contribution percentages >10% were Wuhan flow, city flow, and non-Wuhan flow, with combined contributions (ie, travel-related measures and corresponding measures from previous week) of 49%, 16%, and 12%, respectively. For COVID-19 growth rates, the leading contributing factors were non-Wuhan flow, population density, ILIs, and Wuhan flow, with combined contributions of 26%, 10%, 10%, and 9%, respectively. The remaining factors with contributions of no less than 5% for the growth rate of COVID-19 were city flow (current week and previous week), population number, the number of COVID-19 hospitals, lowest temperature, absolute humidity, and per capita income, with contributions of 7%, 6%, 6%, 6%, 6%, and 5%, respectively (Figure 2).

### City-Level Factor Analysis

**Figure 3:**
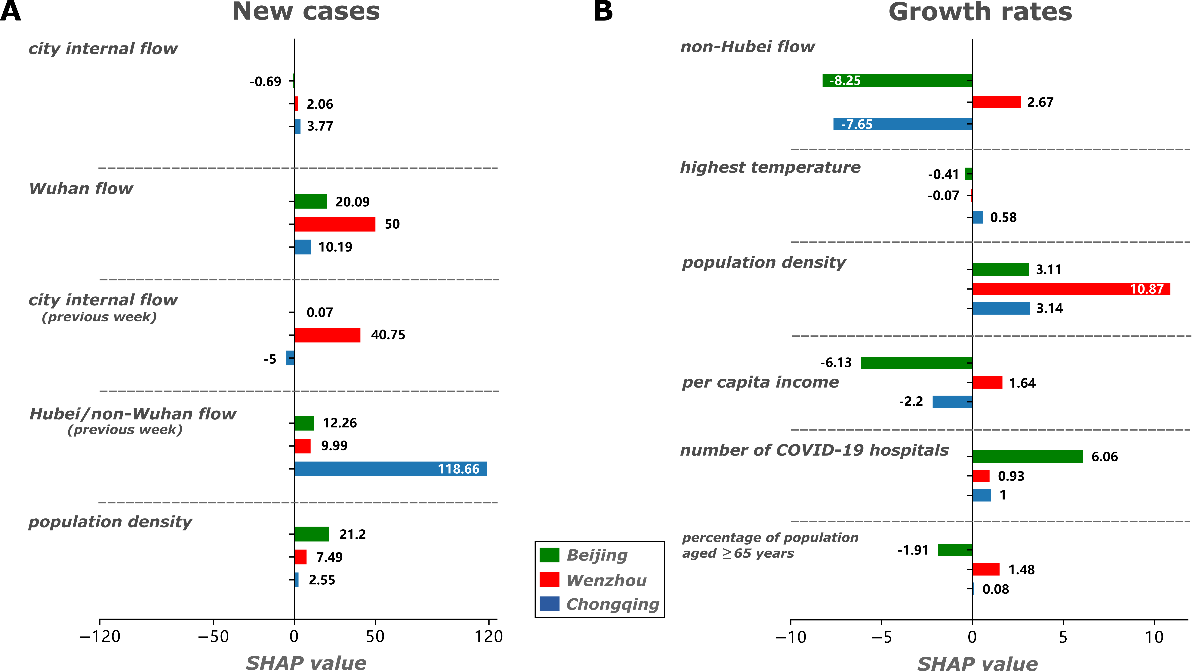
SHAP values for selected factors in Beijing, Wenzhou, and Chongqing. (A) New cases of COVID-19 in the second week. (B) Growth rates of COVID-19 in the first week. Factors were selected by the nonlinear regression tree model. SHAP: Shapely additive explanation.

SHAP is a method for explaining individual predictions [31,35] by computing the contribution of each factor and comparing it to predictions based on game theory. Multimedia Appendix 3 shows the detailed SHAP values of each selected factor for each city. SHAP values explain the contribution of different factors to the COVID-19 outbreak outcomes of each individual city. For a demonstration, we selected a number of new cases from the second week (106, 221, and 179, respectively) and COVID-19 growth rates from the first week (4.08, 36.83, and 6.63, respectively) from Beijing, Wenzhou, and Chongqing (Figure 3). In the second week, Wuhan flow and city internal flow (previous week) contributed to an increased number of new cases in Wenzhou (overall SHAP value, 90.75), while Hubei/non-Wuhan flow was the main factor that led to an increased number of new cases in Chongqing (SHAP value, 118.66) (Figure 3A). For Beijing, population density and Wuhan flow were the top 2 factors that contributed to the number of new cases, with a combined SHAP value of 41.29, which was much smaller than the SHAP values for factors in Wenzhou and Chongqing. This also meant that factors in Beijing related to fewer new cases than factors in Wenzhou and Chongqing (ie, 106 new cases vs 221 and 179 new cases, respectively) (Figure 3A). Furthermore, the differences in COVID-19 growth rates in Wenzhou compared to those in Beijing and Chongqing in the first week (ie, 36.83 vs 4.08 and 6.63, respectively) were caused by population number, non-Hubei flow, per capita income, and the percentage of the population aged ≥65 years (SHAP value: 10.87, 1.67, 1.64, and 1.48, respectively) (Figure 3B). Among them, the contribution from population number was positive for all 3 cities and bigger for Wenzhou, while non-Hubei flow and per capita income were positive for Wenzhou and negative for the other cities, which indicates the importance of those factors to the higher COVID-19 growth rates in Wenzhou. For Beijing, the contribution of the number of COVID-19 hospitals was bigger than in Wenzhou and Chongqing (SHAP value: 6.06 vs 0.93 and 1, respectively) (Figure 3B).

## Discussion

### Principal Results

In this study, we applied a machine learning method to delineate the contribution of different factors to weekly new cases and COVID-19 growth rates based on multidimensional data collected from 343 cities in China. Travel-related factors were the main contributors to the number of new cases and COVID-19 growth rates in China during the outbreak period (ie, January 17 to February 29, 2020), and the growth rate was also affected by other factors, including socioeconomic factors like population density [36]. City-level differences among related factors led to different COVID-19 outbreak outcomes and shed light on targeted prevention and control methods for the future.

The results of our simple correlation analysis and machine learning exploration showed that the progression of the COVID-19 outbreak in China is affected by multiple factors. Based on our nonlinear machine learning method, we found that the most important contributors to new cases and COVID-19 growth rates in China were travel-related factors and that travel-related factors had a clear lag effect that could not be ignored. Previous studies have shown that population movement from Wuhan is the driving factor for new COVID-19 cases in China, and we have confirmed this [2,37,38]. Additionally, we found that population movement from regions beyond Wuhan and internal flow within the city significantly contributed to new cases and COVID-19 growth rates in China, and the population flow from non-Wuhan regions was the leading contributor for COVID-19 growth rates (Figure 2). City internal flow also contributed to the increase in COVID-19 cases and spread of the disease, but its impact was limited. The significant contribution of population movement from non-Wuhan regions emphasizes the importance of monitoring and restricting population movement from regions beyond the disease epicenter during an outbreak. This may relate to the fact that, for people to successfully return to their hometowns and avoid trouble, they might travel in a circuitous manner. Therefore, authorities should pay attention to all travelers, not only the ones directly from Wuhan, especially considering the lag effect of population movement.

Our study also indicated that the contribution of other factors to the progression of the COVID-19 outbreak in China cannot be ignored, especially for the growth rate of COVID-19. The contribution of socioeconomic factors to the growth rate of COVID-19 in China is comparable to that of travel-related factors [39] (Figure 2). The leading contributing socioeconomic factor was population density; a higher population density means a higher probability of secondary infection, resulting in the faster growth of new COVID-19 cases. Among all the factors considered in this study, factors related to medical resources contributed the least to new COVID-19 cases in China, which may indicate that there were enough medical resources for most cities in China. Interestingly, the dominant contributing factor for COVID-19 growth rates among all medical factors was the number of COVID-19 hospitals. This factor had a contribution of 6%, which corresponds to more than 85% of the contribution of all medical factors, indicating that the practice of setting up designated hospitals for COVID-19 in various cities is effective for controlling the growth of cases. Our results also indicated that environmental and ILI factors contributed to COVID-19 growth rates in China, but their contributions were smaller compared to those of travel-related and socioeconomic factors. Therefore, the contribution of environmental and ILI factors should be interpreted carefully and studied further [40,41].

Based on the SHAP values used in game theory, we were able to distinguish the individual contribution of different city-level factors, which has important implications for precise and targeted control strategies. For example, compared to the number of new cases in Beijing, which, as the capital city, is a super megacity with a large population and a hub of population movement, Wenzhou and Chongqing had more new cases in the second week with different contributing factors. Wuhan flow and city internal flow (previous week) were the main contributors for increases in new COVID-19 cases in Wenzhou, while Hubei/non-Wuhan flow (previous week) was the driving factor in Chongqing. To reduce the number of new COVID-19 cases in Wenzhou, efforts beyond restricting population migration from Wuhan are needed, such as reducing the number of social activities within the city. As the adjacent city of Hubei, Chongqing should pay more attention to travelers from Hubei. With regard to the higher COVID-19 growth rates during the first week in Wenzhou, beyond travel-related factors, contributing factors were mainly socioeconomic factors, including population number, per capita income, and percentage of the population aged ≥65 years. Ours is the first study to evaluate the contribution of different city-level factors to outbreak outcomes, and our results and methodology are helpful for the targeted control of infectious diseases.

### Limitations

This study had several limitations. First, although we assessed as many factors as possible, important factors might be missing. For example, although we included measures related to the social distancing policy, such as the city internal flow and city internal flow index, other detailed control policies [42] are missing. Second, due to the multicollinearity among factors and black box effect in the model, our results may not be biologically sound and require careful interpretation. Therefore, negative SHAP values should not be interpreted as factors with a negative effect. Instead, negative SHAP values can indicate a compromised effect and small contribution. Third, more data on medical resources are needed in the future, especially data on the redistribution of medical resources during the epidemic, which are important for effective future resource arrangement. Fourth, although we used a powerful machine learning method to deal with the complex relationships between different factors, it may not the most suitable method. Therefore, other methods should be explored, especially since the data in this study cover a short period of time and exhibit dramatic changes due to strong interventions. Nevertheless, our quantitative results and proposed method shed light on the contribution of different factors to outbreak outcomes and are useful for the precise prevention and control of infectious diseases.

### Conclusions

The prevention and control of the COVID-19 epidemic is a systematical project. Knowing the important contributing factors and prioritizing the corresponding strategies are helpful for creating effective control measures. Beyond population flow from Wuhan, population flow from other places and internal flow within the city also contributed to the number of new cases and COVID-19 growth rates in China. Socioeconomic factors, particularly population number and density, also play very important roles in COVID-19 growth rates in China. The contribution of specific factors for individual cities was also explored based on the framework proposed in this study. The pandemic is still ongoing worldwide, and many countries are experiencing the severe rebound effects of COVID-19. The results we presented and the framework we proposed in this study are helpful and useful for exploring optimal and precise control strategies.

## Appendix

Multimedia Appendix 1 Table S1. A collection of all influencing factors.

Multimedia Appendix 2 Supplementary materials, including Figure S1, Figure S2 and Table S2.

Multimedia Appendix 3 Shapley additive explanation values of each selected factor for each city.

## Abbreviations

ILI: influenza-like illness
SHAP: Shapley additive explanation
XGBoost: extreme gradient boosting
