# Supplementary material for: Impact of Systematic Factors on the Outbreak Outcomes of the Novel COVID-19 Disease in China: Factor Analysis Study
Source: J Med Internet Res. 2020 Nov 11;22(11):e23853. doi: 10.2196/23853 (PMC7661104; doi:10.2196/23853)
Supplement: Multimedia Appendix 2 [file jmir_v22i11e23853_app2.docx]

**Supplementary Materials for**

**Impact of Systematic Factors on the Outbreak Outcomes of the Novel COVID-19 Disease in China：Factor Analysis Study**

Zicheng Cao^1^*, Feng Tang^1^*, Cai Chen^1^, Chi Zhang^1^, Yichen Guo^1^, Ruizhen Lin^1^, Zhihong Huang^1^, Yi Teng^1^, Ting Xie^1^, Yutian Xu^2^, Yanxin Song^3^, Feng Wu^1^, Peipei Dong^1^, Ganfeng Luo^1^, Yawen Jiang^1^, Huachun Zou^1^, Yao-Qing Chen^1^, Litao Sun^1^, Yuelong Shu^1^, Xiangjun Du^1^

1. School of Public Health (Shenzhen), Sun Yat-sen University, Guangzhou, P. R. China
2. School of Intelligent Systems Engineering, Sun Yat-sen University, Guangzhou, P. R. China
3. Lingnan College, Sun Yat-sen University, Guangzhou, P. R. China

* Contributed equally

**Corresponding Author:**

Dr. Xiangjun Du

School of Public Health (Shenzhen), Sun Yat-sen University

Guangzhou, 510006, P. R. China

Phone: +86- 02083226383

Email: duxj9@mail.sysu.edu.cn


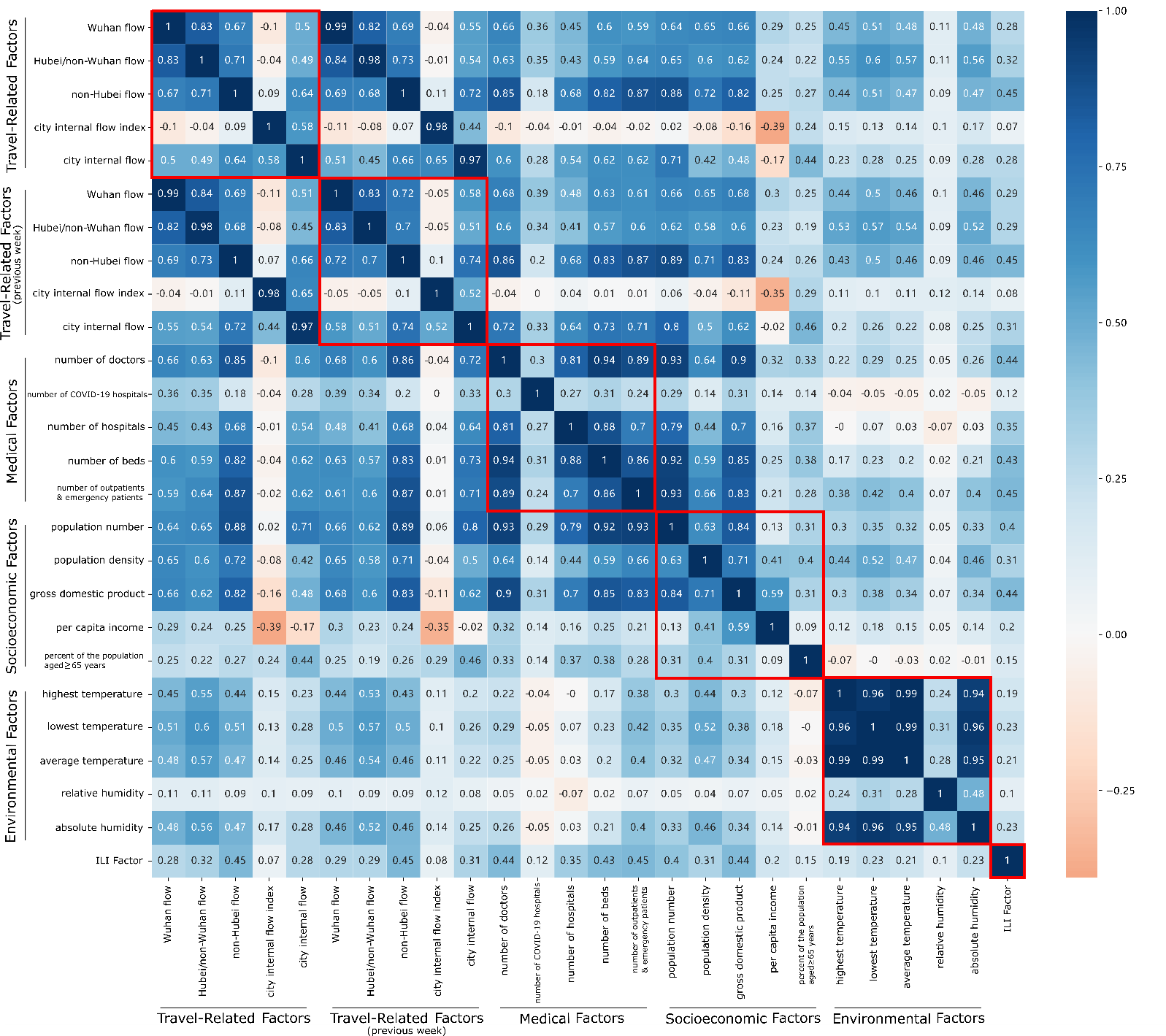


**Figure S1.** Correlation matrix between factors. Spearman correlation coefficients are color coded and factors from the same group are circled.

**
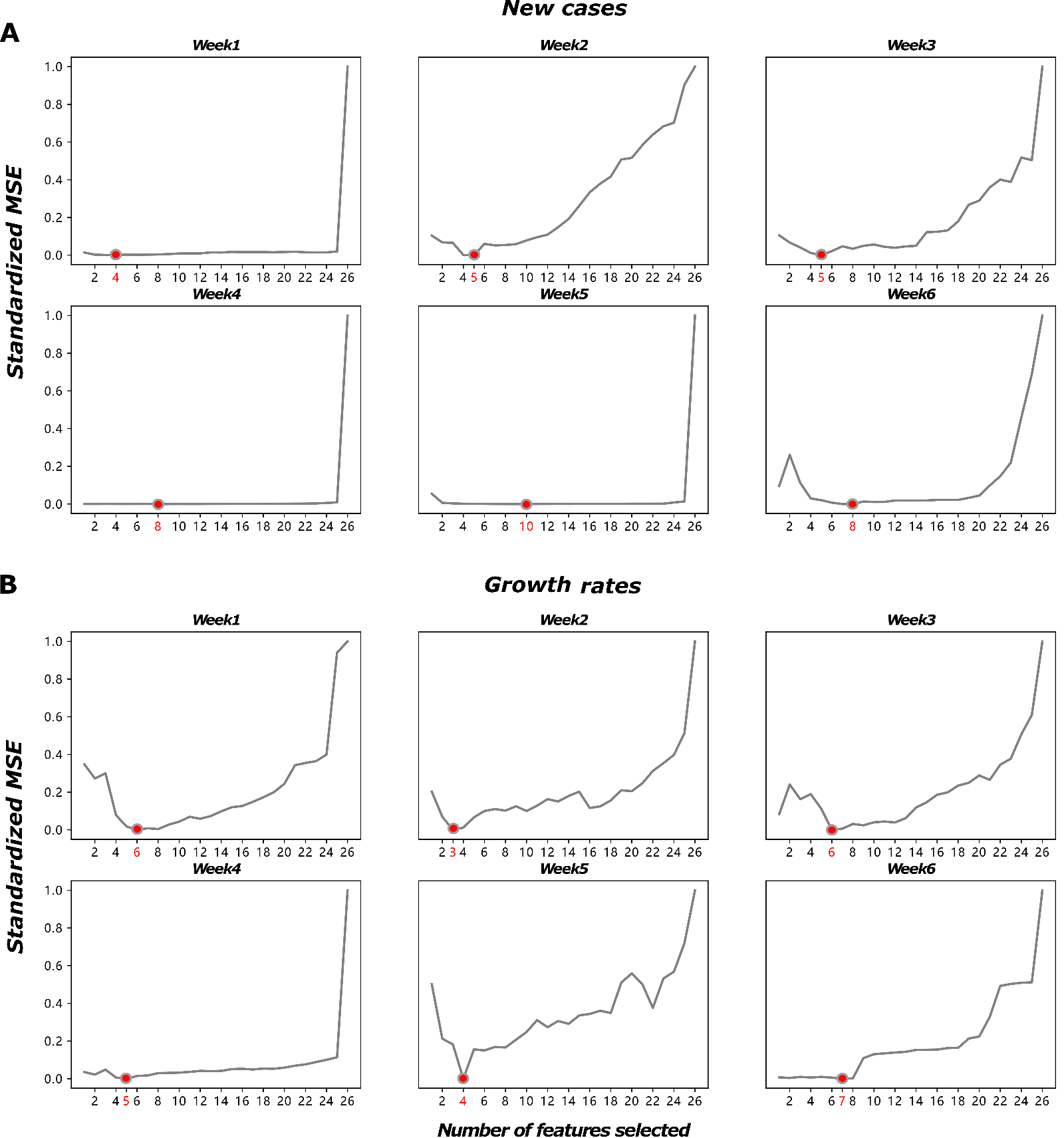
**

**Figure S2: Feature selection curve for new cases (A) and growth rate (B).** The optimal number of features selected is indicated by the red circle. Standardized mean squared error (MSE) was used in this study for selecting important features.

**Table S2. Contribution percentage of factors.** Average contribution percentage is calculated as the mean of the contribution percentage across six weeks. Group contribution percentage is calculated as the sum of the individual contribution for factors within the group and shown at the end of each group.

| Contributing factors | Week1  New cases Growth rates | | Week2  New cases Growth rates | | Week3  New cases Growth rates | | Week4  New cases Growth rates | | | | Week5  New cases Growth rates | | | Week6  New cases Growth rates | | | Average  New cases Growth rates | | |
| --- | --- | --- | --- | --- | --- | --- | --- | --- | --- | --- | --- | --- | --- | --- | --- | --- | --- | --- | --- |
| Wuhan flow | 0 | 0 | 0.45 | 0 | 0.58 | 0.13 | | 0 | | 0 | | 0 | 0.33 | | 0 | 0 | | 0.17 | 0.08 |
| Hubei/non-Wuhan flow | 0 | 0 | 0 | 0 | 0.19 | 0.22 | | 0 | | 0 | | 0 | 0 | | 0 | 0 | | 0.03 | 0.04 |
| non-Hubei flow | 0.20 | 0 | 0 | 0.25 | 0 | 0 | | 0 | | 0 | | 0.04 | 0 | | 0 | 0 | | 0.04 | 0.04 |
| city internal flow index | 0 | 0 | 0 | 0 | 0 | 0 | | 0 | | 0 | | 0 | 0 | | 0 | 0 | | 0 | 0 |
| city internal flow | 0 | 0 | 0.12 | 0 | 0 | 0 | | | 0 | 0.25 | | 0.10 | 0 | | 0.28 | 0 | | 0.08 0.04 | |
| **Travel-Related Factors** | **0.20** | **0** | **0.57** | **0.25** | **0.77** | **0.35** | | **0** | | **0.25** | | **0.14** | **0.33** | | **0.28** | **0** | | **0.32** | **0.20** |
|  |  |  |  |  |  |  | |  | |  | |  |  | |  |  | |  |  |
| Wuhan flow | 0.49 | 0 | 0 | 0 | 0.13 | 0 | | | 0.31 | 0.08 | | 0.49 | 0 | | 0.51 | 0 | | 0.32 0.01 | |
| Hubei/non-Wuhan flow | 0 | 0 | 0.22 | 0 | 0 | 0.14 | | 0 | | 0.21 | | 0.06 | 0 | | 0 | 0.16 | | 0.05 | 0.09 |
| non-Hubei flow | 0 | 0.29 | 0 | 0 | 0 | 0.13 | | | 0 | 0 | | 0 | 0 | | 0 | 0.13 | | 0 0.09 | |
| city internal flow index | 0 | 0 | 0 | 0 | 0 | 0 | | | 0.32 | 0 | | 0 | 0 | | 0 | 0.11 | | 0.05 0.02 | |
| city internal flow | 0 | 0 | 0.09 | 0 | 0.06 | 0 | | 0 | | 0 | | 0 | 0 | | 0 | 0.03 | | 0.03 | 0.01 |
| **Travel-Related Factors (previous week)** | **0.49** | **0.29** | **0.31** | **0** | **0.19** | **0.27** | | **0.63** | | **0.29** | | **0.55** | **0** | | **0.51** | **0.43** | | **0.45** | **0.21** |
|  |  |  |  |  |  |  | |  | |  | |  |  | |  |  | |  |  |
| number of doctors | 0 | 0 | 0 | 0 | 0 | 0 | | 0 | | 0 | | 0 | 0 | | 0.03 | 0.06 | | 0.01 | 0.01 |
| number of COVID-19 hospitals | 0 | 0.13 | 0 | 0 | 0 | 0 | | 0.07 | | 0 | | 0 | 0.20 | | 0 | 0 | | 0.01 | 0.06 |
| number of hospitals | 0.12 | 0 | 0 | 0 | 0 | 0 | | 0 | | 0 | | 0 | 0 | | 0 | 0 | | 0.02 | 0 |
| number of beds | 0 | 0 | 0 | 0 | 0 | 0 | | 0 | | 0 | | 0.04 | 0 | | 0 | 0 | | 0.01 | 0 |
| number of outpatients & emergency patients | 0 | 0 | 0 | 0 | 0 | 0 | | 0 | | 0 | |  | 0 | | 0 | 0 | | 0 | 0 |
| **Medical Factors** | **0.12** | **0.13** | **0** | **0** | **0** | **0** | | **0.07** | | **0** | | **0.04** | **0.20** | | **0.03** | **0.06** | | **0.05** | **0.07** |
|  |  |  |  |  |  |  | |  | |  | |  |  | |  |  | |  |  |
| population number | 0 | 0.13 | 0 | 0 | 0 | 0 | | 0.09 | | 0 | | 0.10 | 0.21 | | 0 | 0 | | 0.03 | 0.06 |
| population density | 0 | 0 | 0.13 | 0 | 0 | 0.26 | | 0 | | 0 | | 0.05 | 0 | | 0.09 | 0.31 | | 0.05 | 0.10 |
| gross domestic product | 0 | 0 | 0 | 0 | 0 | 0 | | 0.05 | | 0 | | 0.01 | 0.26 | | 0 | 0 | | 0.01 | 0.04 |
| per capita income | 0 | 0.20 | 0 | 0 | 0 | 0.13 | | 0 | | 0 | | 0 | 0 | | 0 | 0 | | 0 | 0.05 |
| percentage of the population aged ≥65 years | 0 | 0.06 | 0 | 0 | 0 | 0 | | 0.01 | | 0.13 | | 0 | 0 | | 0 | 0 | | 0 | 0.03 |
| **Socioeconomic Factors** | **0** | **0.39** | **0.13** | **0** | **0** | **0.39** | | **0.15** | | **0.13** | | **0.16** | **0.47** | | **0.09** | **0.31** | | **0.09** | **0.28** |
|  |  |  |  |  |  |  | |  | |  | |  |  | |  |  | |  |  |
| highest temperature | 0 | 0.18 | 0 | 0 | 0 | 0 | | 0 | | 0 | | 0 | 0 | | 0 | 0 | | 0 | 0.03 |
| lowest temperature | 0 | 0 | 0 | 0.35 | 0 | 0 | | 0 | | 0 | | 0 | 0 | | 0.01 | 0 | | 0 | 0.06 |
| average temperature | 0 | 0 | 0 | 0 | 0 | 0 | | 0 | | 0 | | 0 | 0 | | 0 | 0 | | 0 | 0 |
| relative humidity | 0.19 | 0 | 0 | 0 | 0 | 0 | | 0 | | 0 | | 0.10 | 0 | | 0.08 | 0 | | 0.06 | 0 |
| absolute humidity | 0 | 0 | 0 | 0 | 0.03 | 0 | | 0.06 | | 0.33 | | 0 | 0 | | 0 | 0 | | 0.02 | 0.06 |
| **Environmental Factors** | **0.19** | **0.18** | **0** | **0.35** | **0.03** | **0** | | **0.06** | | **0.33** | | **0.10** | **0** | | **0.09** | **0** | | **0.08** | **0.14** |
|  |  |  |  |  |  |  | |  | |  | |  |  | |  |  | |  |  |
| ILI | 0 | 0 | 0 | 0.41 | 0 | 0 | | 0.09 | | 0 | | 0 | 0 | | 0 | 0.20 | | 0.01 | 0.1 |
| **ILI Factor** | **0** | **0** | **0** | **0.41** | **0** | **0** | | **0.09** | | **0** | | **0** | **0** | | **0** | **0.20** | | **0.01** | **0.1** |
